# Developing 3D SEM in a broad biological context

**DOI:** 10.1111/jmi.12211

**Published:** 2015-01-26

**Authors:** A Kremer, S Lippens, S Bartunkova, B Asselbergh, C Blanpain, M Fendrych, A Goossens, M Holt, S Janssens, M Krols, J-C Larsimont, C Mc Guire, MK Nowack, X Saelens, A Schertel, B Schepens, M Slezak, V Timmerman, C Theunis, R Van Brempt, Y Visser, CJ GuÉRin

**Affiliations:** * VIB Bio Imaging Core, Gent, VIBTechnologiepark 927, Gent, B-9052, Belgium; † Inflammation Research Center, VIBTechnologiepark 927, Gent, B-9052, Belgium; ‡ Department of Biomedical Molecular Biology, Ghent UniversityGhent, Belgium; § VIB Department of Molecular Genetics, Antwerp UniversityAntwerpen 2020, Belgium; || IRIBHM, Université Libre de BruxellesBrussels, B-1070, Belgium; # Department of Plant Systems Biology, VIBGhent, 9052, Belgium; ** Department of Plant Biotechnology and Bioinformatics, Ghent University9052, Ghent, Belgium; †† Institute of Science and Technology (IST) AustriaKlosterneuburg, 3400, Austria; ‡‡ Center for the Biology of Disease, VIBLeuven, Belgium; §§ Institute for Biology/Genetics, Freie Universität BerlinBerlin, Germany; |||| Department of Respiratory Medicine, Ghent UniversityGhent, Belgium; ## GROUP-ID Consortium, Ghent University and University HospitalGhent, Belgium; *** Carl Zeiss Microscopy, GmbHOberkochen, Germany; ††† Department of Intensive Care, Leiden University Medical CenterLeiden, The Netherlands; ‡‡‡ Johnson and Johnson Pharmaceutical Research and DevelopmentBeerse, Belgium

**Keywords:** Correlative light and electron microscopy, focused ion beam scanning electron microscopy, sample preparation, serial block-face scanning electron microscopy

## Abstract

When electron microscopy (EM) was introduced in the 1930s it gave scientists their first look into the nanoworld of cells. Over the last 80 years EM has vastly increased our understanding of the complex cellular structures that underlie the diverse functions that cells need to maintain life. One drawback that has been difficult to overcome was the inherent lack of volume information, mainly due to the limit on the thickness of sections that could be viewed in a transmission electron microscope (TEM). For many years scientists struggled to achieve three-dimensional (3D) EM using serial section reconstructions, TEM tomography, and scanning EM (SEM) techniques such as freeze-fracture. Although each technique yielded some special information, they required a significant amount of time and specialist expertise to obtain even a very small 3D EM dataset. Almost 20 years ago scientists began to exploit SEMs to image blocks of embedded tissues and perform serial sectioning of these tissues inside the SEM chamber. Using first focused ion beams (FIB) and subsequently robotic ultramicrotomes (serial block-face, SBF-SEM) microscopists were able to collect large volumes of 3D EM information at resolutions that could address many important biological questions, and do so in an efficient manner. We present here some examples of 3D EM taken from the many diverse specimens that have been imaged in our core facility. We propose that the next major step forward will be to efficiently correlate functional information obtained using light microscopy (LM) with 3D EM datasets to more completely investigate the important links between cell structures and their functions.

**Lay Description:**

Life happens in three dimensions. For many years, first light, and then EM struggled to image the smallest parts of cells in 3D. With recent advances in technology and corresponding improvements in computing, scientists can now see the 3D world of the cell at the nanoscale. In this paper we present the results of high resolution 3D imaging in a number of diverse cells and tissues from multiple species. 3D reconstructions of cell structures often revealed them to be significantly more complex when compared to extrapolations made from 2D studies. Correlating functional 3D LM studies with 3D EM results opens up the possibility of making new strides in our understanding of how cell structure is connected to cell function.

## Introduction

Seeing into the world of the cell through the use of magnifying devices has fascinated scientists and nonscientists alike since Antonie van Leeuwenhoek in the 17th century used his simple microscope to see the microworld of the cell (Hoole, 1800). Microscopes have developed over the centuries and today microscopy remains an essential technique in the Life Sciences. Microscopy has contributed to observations on every scale: from the first observation of single-celled bacteria by van Leeuwenhoek through the detailed anatomy of the mammalian brain by Camillo Golgi and Ramon Y Cajal (Golgi, 1873; Ramon y Cajal, 1890), and more recently the live imaging of intracellular organelles and protein complexes (Cognet *et al*., 2014). However, there is a limit to the use of photons for microscopic imaging, which was first calculated by Ernst Abbe in 1873 (Abbe, 1873). By Abbe’s theory the ultimate resolution of a light microscope in *X*,*Y* is 187 nm and that can only be achieved with the use of high-quality objective lenses and thin specimens. This is problematic since developments in molecular biology have revealed the importance of protein function and dissecting molecular pathways, highlighting the need to image increasingly small structures. Clever methods have been developed to circumvent the Abbe diffraction limit in light microscopy (LM), the variously named super-resolution techniques (Saka & Rizzoli, 2012; Swedlow, 2012). These techniques still have their limits, generally becoming ineffective below 50 nm. To answer certain biological questions we require better resolution than that.

Though LM continues to develop, it is unlikely to reach resolutions where cellular ultrastructure becomes visible. For that we can make use of electrons. Electrons as a means of imaging structures below LM limits came out of the wave-particle theory developed by De Broglie in 1924 (de Broglie, 1925a, b). Just 2 years later, the first electromagnetic lenses were developed by Hans Busch (Busch, 1927), allowing Ernst Ruska together with Max Knoll to construct a prototype TEM in 1931 (Ruska *et al*., 1940). In TEM, electrons are propelled through a thin tissue-section (typically 50–90 nm), extending resolution to the Å range. In a scanning electron microscope, developed by Max Knoll in 1933, the electron beam scans the surface of a sample and is able to resolve structures in the nm range. Using these techniques we are able to see the smallest structures in the cell. However, the requirement of electron microscopes to operate under high vacuum and their strong electron beam precludes any experiments with living samples. Further removing EM from the world of the living cell was the fact that the technique inherently resulted in 2D images, either from the thin sections needed for TEM or the surface imaging only nature of SEM. So although resolution limits were not an issue in EM it came at the expense of 3D visualization.

The goal of biological microscopy is to image life in its most natural form. Life happens in three dimensions and 3D EM imaging has been slow to develop. 3D LM was greatly enhanced with the introduction of the confocal microscope, which allowed highly resolved optical sections to be collected and reconstructed in 3D (Cremer & Cremer, 1978; Sheppard & Kompfner, 1978). In electron microscopy (EM), imaging in 3D was initially dependent on serial-section TEM or electron tomography (ET) (Linberg & Fisher, 1986; Knott *et al*., 2009; Mishchenko, 2009; Bock *et al*., 2011; Takemura *et al*., 2013). These techniques are very labour intensive and require a high degree of skill and training. Electron tomography has been yielding high-resolution 3D data for over 40 years (Hoppe, 1974). Using this method a “thick” (up to 500 nm) sample is placed in a high-voltage TEM and imaged at increasing angles around the centre of the sample. 3D views can be reconstructed from the resulting image sequences (McEwen & Marko, 2001; Subramaniam *et al*., 2003). Still, the depth that can be imaged is limited, it requires very expensive equipment (a high-voltage TEM) and it is very labour-intensive. Serial section TEM did produce volume information but in the predigital imaging era there was a limit to what could be done with that information, in particular with respect to reconstructions. Bock *et al*., used the power of digital imaging to collect and reconstruct data from 1215 serial sections of brain in an attempt to determine the connectivity in the visual cortex (Bock *et al*., 2011). In a subsequent study Takemura *et al*. acquired more than 200,000 micrographs resulting in the reconstruction of 379 neurons (Takemura *et al*., 2013). Even though they developed a semiautomated reconstruction workflow, this study demonstrates that serial sectioning and manually imaging the sections is a very labour-intense method. Another technique which has been used to bring the third dimension to EM, is array tomography where serially sectioned cells or tissues are mounted on silicon wafers in exact order and imaged in an SEM (Hayworth *et al*., 2014). SEMs generally provide surface information, but if a surface is perfectly smooth, backscattered electrons can be used to obtain information from the first few nanometres below the surface, resulting in TEM like images. Automatically cutting and then scanning ribbons of serial sections reduces the time needed for imaging in 3D EM considerably, but is still not devoid of a significant and time-consuming technical input. Although all these methods have brought 3D EM imaging none are easy or efficient to perform and as a result publications using these approaches are few.

Over the last 20 years, development of two new methods has led to a dramatic increase in 3D EM studies (Peddie & Collinson, 2014). The first, serial block-face imaging SEM (SBF-SEM), developed by Winfried Denk, uses an automated ultramicrotome located in the SEM chamber (Denk & Horstmann, 2004). The second uses a focused ion or plasma beam (FIB) to mill away a thin section of a hard substrate also done in the SEM chamber (Young *et al*., 1993; Bushby *et al*., 2011). Both methods are based upon the principle of block-face imaging, that is, that the surface of a plastic embedded block of cells or tissue is imaged then sectioned and reimaged. The process can run in an automated manner to collect many hundreds of serial images. Using the SEM at low electron energies the depth of electron imaging is limited to the upper few nm of the block-face, ensuring high *Z* resolution (Briggman & Bock, 2012). These new technologies have brought the full 3D to nanoscale imaging while also delivering efficiency and high-quality results.

The primary difference in these new methods is in their method of sectioning. In SBF-SEM, sectioning is performed by an automated ultramicrotome located in the SEM chamber, automatically removing thin sections (≥20 nm thick) from the block-face (Denk & Horstmann, 2004). Making use of a very high-resolution detector (the Gatan 3View is equipped with a detector that allows acquisition of images up to 32K × 24K pixels) ensures that relatively large areas (up to 500 μm^3^) can be scanned at high *X*, *Y* resolution, providing both large overviews and detailed ultrastructure (Holcomb *et al*., 2013). The resulting 3D images will consist of nonisotropic voxels, because the *X*, *Y* resolution is smaller than the *Z* slicing. One important consideration is that one image, or one ‘slice’ contains information of the first nanometres below the sample surface due to the low voltages used in acquisition, whereas slicing is done at tens of nanometres.

In FIB-SEM the FIB propels Gallium ions towards the block-face, which at high energies results in the milling of the sample surface, removing sections as thin as 5 nm. Subsequent milling and imaging of the block-face, results in image stacks with the possibility of very small isotropic voxels providing the data for precise high-resolution 3D reconstructions, albeit with limited total volume, approximately 50 μm^3^ (Ballerini *et al*., 2001). Although SEM is not capable of achieving the levels of resolution seen in TEM, the resolution obtained with modern field emission SEM technology (FE-SEM) (<2 nm) allows us to answer many biological questions. Using these techniques, stacks of thousands of serial images have been acquired for 3D reconstructions (Denk & Horstmann, 2004; Knott *et al*., 2011; Holcomb *et al*., 2013; Maco *et al*., 2013; Starborg *et al*., 2013; Hughes *et al*., 2014; Peddie & Collinson, 2014), making these extremely powerful tools in the Life Sciences.

Bringing together the data from live sample functional LM with 3D SEM is our ultimate goal. Correlating functional imaging in LM with the ultra-structural detail at specific time-points to elucidate the exact processes at an ultrastructural level will open up new and powerful areas of experimentation. The concept of such correlative light and electron microscopy (CLEM) was first used in the 1960s (Godman *et al*., 1960; Abandowitz & Geissinger, 1975). A plethora of different combinations of LM and EM techniques were used since then for CLEM; from bright-field or fluorescence imaging correlating with classical SEM, to live confocal imaging of dendritic spines in mouse brain correlated with serial sectioning TEM or FIB-SEM, but also combining light and EM with μCT/X-ray imaging (Heymann *et al*., 2006; Knott et al., 2009; Robinson & Takizawa, 2009; Caplan *et al*., 2011; Muller-Reichert & Verkade, 2012; Handschuh *et al*., 2013; Arkill *et al*., 2014; Maco *et al*., 2014). In light of the recent advances in 3D imaging on both the LM and EM scale we strongly believe that the future of CLEM will rest upon high-resolution 3D imaging in both modalities. The combination of LM and EM in 3D is the next step that will help link the functional and structural details of cells and tissues.

## Materials and methods

### SBF-SEM

For SBF-SEM, samples were fixed and prepared with variations of the protocol as described by Deerinck *et al*. (2010). Resin embedded samples were mounted on an aluminium specimen pin (Gatan, www.gatan.com), using conductive epoxy (Circuit Works, www.chemtronics.com). The specimens were precision trimmed in a pyramid shape using an ultramicrotome and coated with 5 nm of Pt, in a Quorum Q 150T ES sputter coater (www.quorumtech.com). The aluminium pins were placed in the Gatan 3View2 (www.gatan.com) in a Zeiss Merlin SEM (www.zeiss.com). The block was faced with the 3View ultramicrotome unit to remove the platinum top layer and for imaging we used the Gatan Digiscan II ESB detector at an accelerating voltage of between 1.3 and 1.8 kV (sample dependent).

An important adaptation to the staining protocol for Arabidopsis root tips and other fragile samples was an agarose embedding step after the initial fixation, and for plants the use of ruthenium red and Spurr’s epoxy. In brief, 5-day-old Arabidopsis seedlings on agar plates were fixed in 0.1 M phosphate buffer pH 6.8, 3% glutaraldehyde and 2% paraformaldehyde for 2 h. Individual samples were encased in rectangular agarose blocks, as described (Wu *et al*., 2012). The samples were transferred to fresh fixative and kept overnight at 4 °C. The next day, samples were washed 5× 3 min in cold 0.15 M cacodylate buffer. E*n bloc* contrast staining was performed by consecutive incubations in heavy metal containing solutions. Between these steps samples were always washed 5× 3 min in ultrapure water (UPW). The first staining step was a 1-h incubation on ice in 0.2% ruthenium red and 2% aqueous osmium tetroxide in 0.15 M cacodylate buffer. After washing, the samples were incubated for 20 min in a fresh thiocarbohydrazide solution (1% w/v in UPW) at room temperature (RT). The next wash step was followed by incubation in 2% osmium in UPW at RT for 30 min and 2% uranyl acetate at 4 °C overnight. The following day, Walton’s lead aspartate staining was performed for 30 min at 60 °C. For this, a 30 mM L-aspartic acid solution was used to freshly dissolve lead nitrate (20 mM, pH 5.5), the solution was filtered and blocks incubated for 30 min at 60 °C. After final washing steps, the samples were dehydrated using ice-cold solutions of 30%, 50%, 70%, 90%, 2× 100% ethanol (anhydrous), 2× 100% aceton, 30 min each. Resin embedding was done using Spurr’s (Electron Microscopy Sciences, www.electronmicroscopysciences.com/) by first placing the samples in 30% propylene oxide/Spurr’s for 2 h, 50% propylene oxide/Spurr’s for 2 h, followed by three incubations in 100% Spurr’s (overnight, 8 h and overnight). The next day samples were put in fresh Spurr’s resin and placed at 60 °C for 24 h. The use of Spurr’s as embedding resin was also preferred for skin tissue due to its low viscosity. When making use of lanthanide salts, staining was performed as described (Deerinck *et al*., 2010), replacing the uranyl acetate incubation step with overnight incubation in UAR-EMS uranyl acetate replacement stain (Electron Microscopy Sciences, www.electronmicroscopysciences.com) 1:3 in H_2_O.

### FIB-SEM

For FIB-SEM, cellular monolayers were grown either on Aclar (7.8 mils, Electron Microscopy Sciences, www.electronmicroscopysciences.com) or on gridded coverslips (MatTek Corporation, 200 Homer Ave, Ashland, MA 01721, USA). Samples were prepared as described by Knott *et al*. (2011) and resin embedded samples were mounted on stubs and coated with 8 nm of Pt in a Quorum Q 150T ES sputter coater (Quorum Technologies, www.quorumtech.com). The stubs were placed in the Zeiss Auriga FE-FIB-SEM (www.zeiss.com) and the SEM was used at 15 kV to visualize cells/tissues below the Pt layer and localize the cell or region of interest (ROI). Subsequently, the stage was tilted 54°, so that the surface was perpendicular to the FIB. Using the gas injection system (GIS) an additional layer of Pt was deposited on the ROI to protect the surface from beam damage (FIB current 500pA-1nA, 5 min). To create a surface to image with the SEM, a trench was milled just before the Pt covered area, using high currents (6.5–10 nA) to reduce the preparation time. After this, the sample was allowed to stabilize in the vacuum for at least 1 h, but preferably overnight to minimize drift during imaging. Images were acquired using an ESB detector (grid: 1194 V) at 1.5 kV and 3072 × 2304 pixels. Imaging parameters were adjusted so that imaging time did not exceed 1 min. Next, the FIB was set up to mill the Pt covered area at a current of 2 nA and 5–20 nm sections.

### Microwave tissue processing

Tissue samples from mouse brain and skin were fixed in 2% PFA, 2.5% glutaraldehyde in 0.15 M Cacodylate buffer pH 7.4 with 2 mM CaCl_2_. Samples of Arabidopsis leaves were fixed in 0.5% PFA, 2.5% Glutaraldehyde in 0.1 M phosphate buffer pH 6.8. After several washes in buffer samples were processed in a Pelco Biowave Pro, (Ted Pella, Inc., www.tedpella.com) with use of microwave energy and vacuum. Briefly, samples were fixed in 1% reduced Osmium in cacodylate buffer with CaCl_2_ 7× 2 min with alternating microwave power of 100 W/0 W. This step was repeated once. After two washes in UPW with power of 250 W, samples were stained in 1% Uranyl acetate 7× 1 min with alternating microwave power of 150 W/0 W. After two washes in UPW, samples were dehydrated in series of EtOH, each step 40 s at 250 W without vacuum. In the next steps samples were infiltrated in series of different dilutions of Epon resin: EtOH 7× 3 min each at 250 W with vacuum. Finally, samples were embedded in Epon resin 30 min at 200 W, 2× 45 min at 375 W with no vacuum.

### Image processing

Images were acquired as a series of 2D tiff (FIB-SEM) or dm3 (3view) files. In order to compile a 3D tiff file format, the images were registered in Fiji (Schindelin *et al*., 2012) (http://fiji.sc/Fiji; Plugin Registration > StackReg > Translation) or IMOD (Kremer *et al*., 1996) (http://bio3d.colorado.edu/imod/; tiltxcorr algorithm). Representation of orthogonal views and/or threshold-based segmentation was done in Imaris (BitPlane, www.bitplane.com) or Fiji’s 3D Viewer. Manual segmentation and visualization of 3D data was done using 3DMOD (http://bio3d.colorado.edu/imod/doc/3dmodguide.html).

3D reconstructions of confocal images were done using Volocity (PerkinElmer, www.perkinelmer.co.uk/volocity).

### Near infrared branding

After live-imaging, mouse brain samples were fixed for 3D EM using 2% PFA, 2.5% GA in 0.15 M cacodylate buffer. Subsequently, one hemisphere was sectioned at 60 μm using a Leica VT1200S vibratome (Leica, www.leica-microsystems.com), and the ROI was reacquired using the pattern of blood vessels visualized with phase-contrast LM as a guide. After determining which section contained the region imaged by LM, four small laser brands were scarred into the tissue using the NIRB technique as described by Bishop *et al*. (2011) using a Zeiss LSM 780 (www.zeiss.com) equipped with a Spectra Physics MaiTai multiphoton laser. Tissue was subsequently prepared for EM.

## Results

### Volume scanning electron microscopy in diverse samples

**Figure 1:**
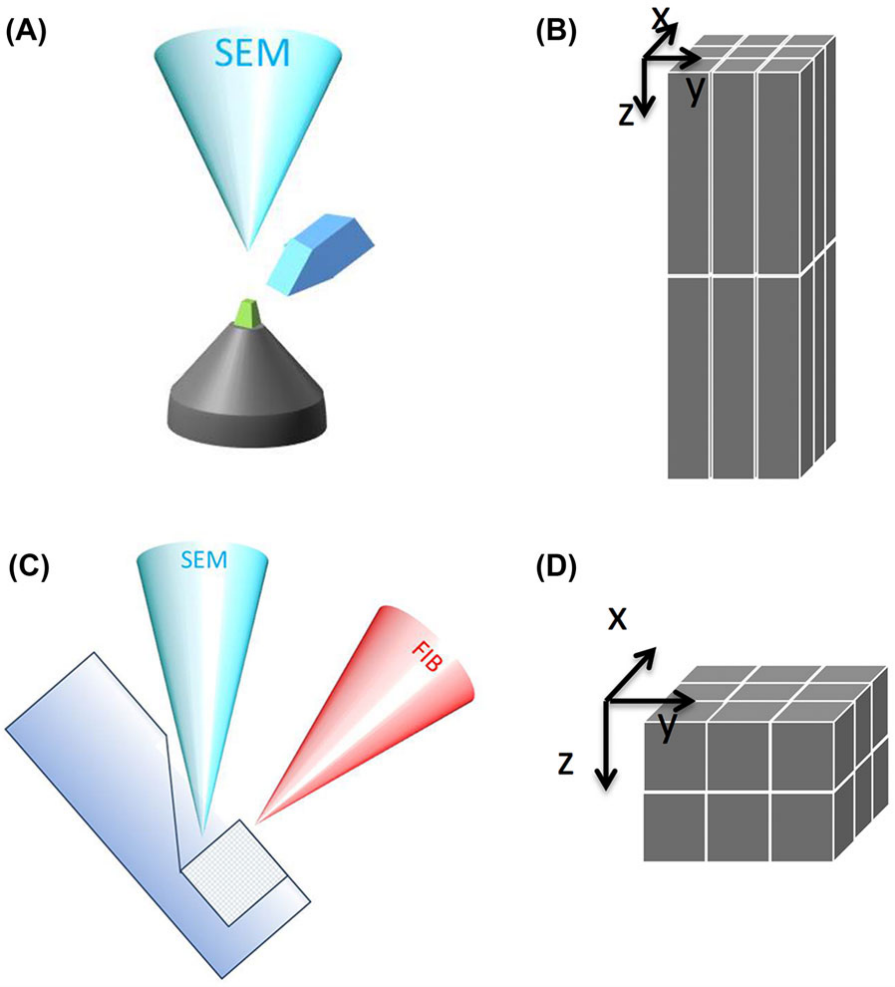
Schemes of serial block-face SEM techniques. (A) A schematic view of SBF-SEM: the electron beam scans the surface of the sample and sections (≥20 nm) are removed by a fully automated ultra-microtome. (B) Images acquired by SBF-SEM results in voxels with a larger *z* than *x*, *y* dimensions. (C) A schematic view of FIB-SEM: a Focused Ion Beam aligned to a coincidence point with the electron column (SEM) is used to create a surface for imaging with the electron beam, and subsequently to mill away sections ≥5 nm. (D) Thin sections in the FIB-SEM allow imaging at isotropic voxels, where *x* = *y* = *z*.

**Figure 2:**
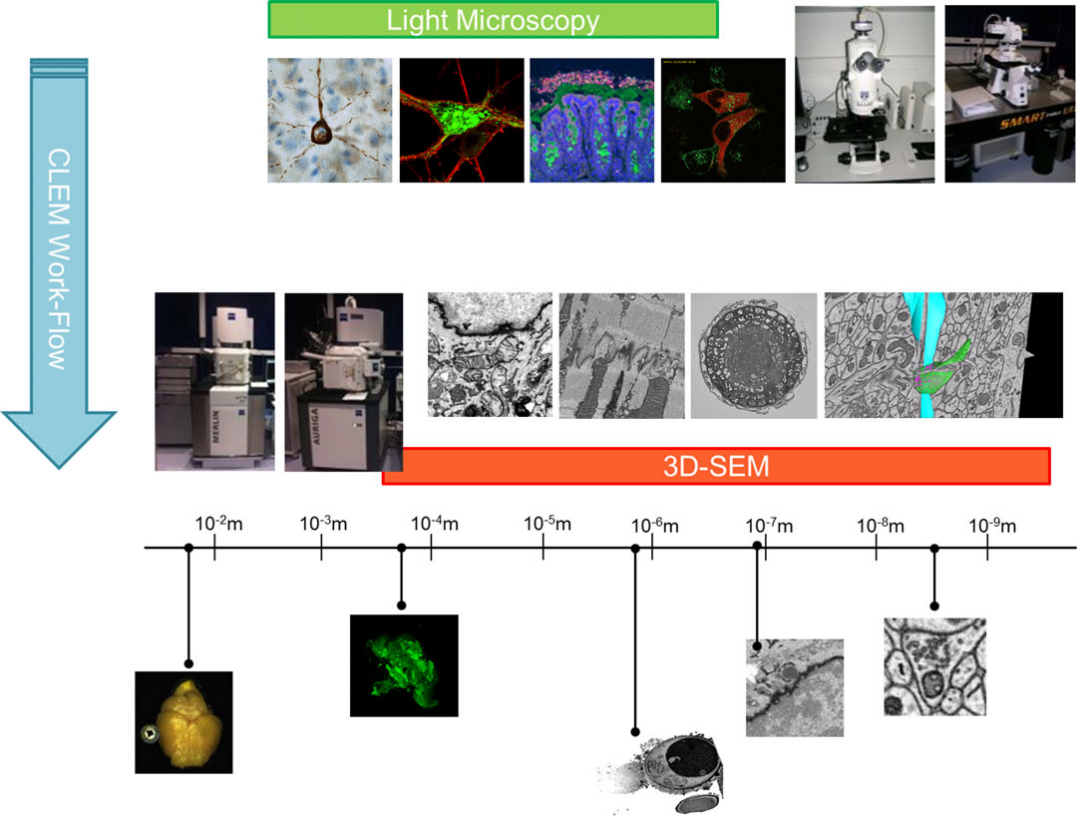
Summary of imaging modalities and scales. Diagram illustrating the imaging modalities and scales used in correlative microscopy. Making use of LM techniques that can yield large fields of view as well as high resolution allows for functional studies to be performed. Methods for preserving, preparing and transferring these samples to 3D SEM can be used to create a correlative microscopy work flow to better link cellular events to the underlying cellular nano-structure.

We have successfully imaged numerous samples with both 3D SEM methods (Figs.1 and 2, Table1) including many different tissue and species types. These different sample types have each required optimization for 3D SEM and there is no one-size-fits-all protocol that can be applied if optimal results are to be achieved.

**Table 1:** List of samples

Bacteria
*Pichia pastoris*
Entire organism
*Arabidopsis thaliana*
Root tip
*Medicago truncatula*
Root tip
Stem
*Drosophila melanogaster*
Ventral nerve cord
Adult brain
*Mus musculus*
Brain
Choroid plexus
Heart
Lung
Pancreas
Skin
Cultured mouse embryonic fibroblasts
*Homo sapiens*
Cultured HeLa cells

All examples of 3D SEM images that are shown in this paper were acquired with a Zeiss Merlin or Zeiss Auriga SEM. The resolution of the microscopes is, for the settings used, between 1 and 2 nm. When we mention voxels sizes for the presented 3D images, we refer to pixel size for *X*, *Y* and to the slice thickness for *Z*.

### Imaging using SBF-SEM and FIB-SEM

**Figure 3:**
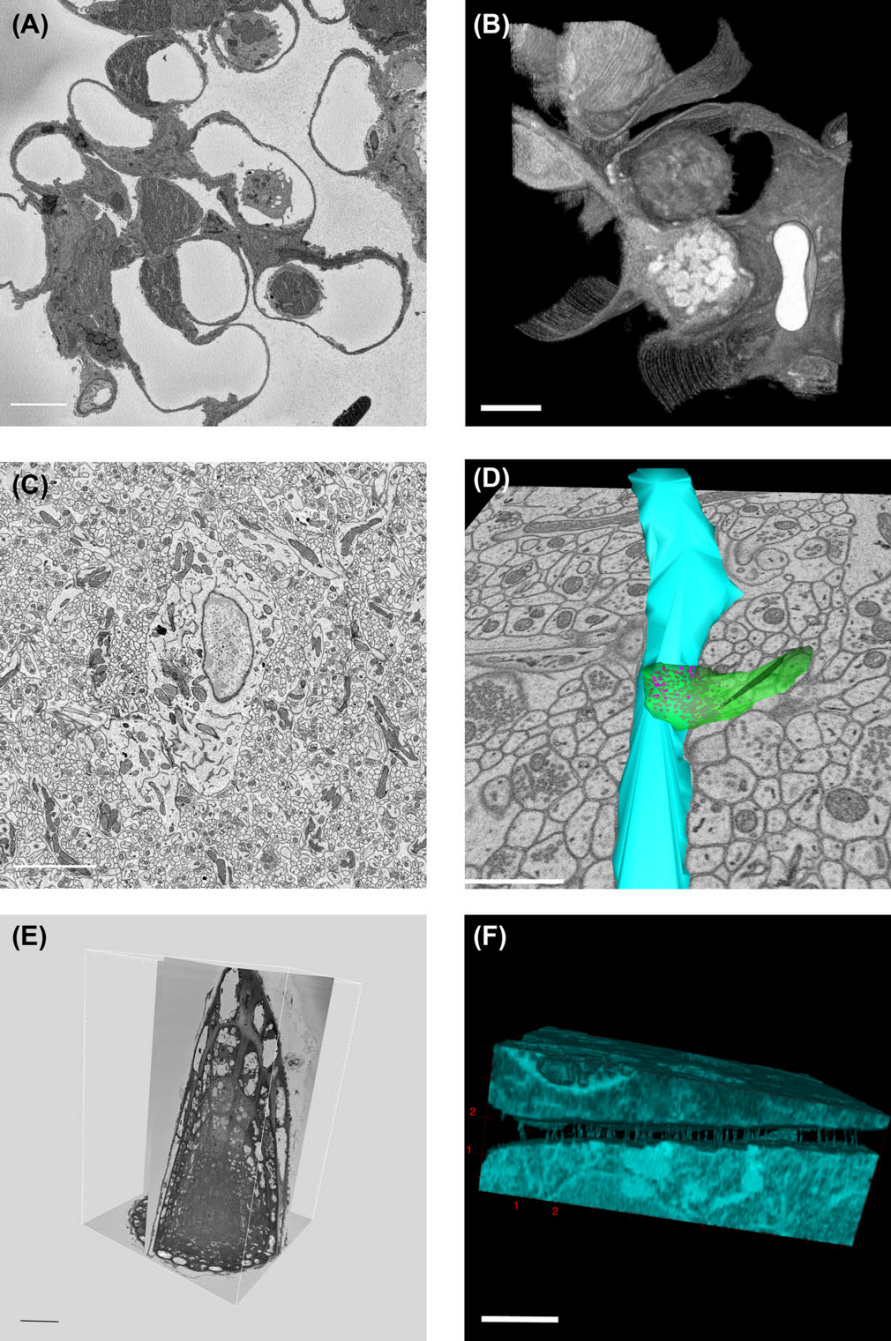
3D-EM images, acquired by SBF-SEM. (A) Mouse lung tissue imaged by SBF-SEM, *X*, *Y* pixels 37 nm, *Z* slices 70 nm. Single plane from 3D series showing lung alveoli and immune cells. Dark areas in left corners are caused by charging, a common issue when imaging bare resin. Scale bar: 5 μm. (B) Volume representation of the image stack made in Fiji. This view shows an immune cell residing in an alveolus. Total volume = 19,47 × 22,42 × 7,14 μm. Scale bars 5 μm. (C) Mouse brain imaged at 4000 × 4000 pixels, *X*, *Y* pixels 6.3 × 6.3 nm *Z* slices 40 nm. Scale bar: 5μm. (D) Manual segmentation of a subset (7,91 × 7,51 × 0,84 μm) of this dataset done using IMOD. Dendrites are presented in blue, a synapse in green and its synaptic vesicles in pink. Scale bar: 1μm. (E) Orthogonal views of 2000 slices at 75 nm *Z* of a 5-days-old seedling from *Arabidopsis thaliana*. Scale bar: 20 μm. (F) *Arabidopsis* root tip image stack of 10,35 × 7,23 × 3,9 μm. 3D volume reconstruction shows plasmodesmata that connect the cytoplasm of 2 neighboring cells. Scale bar: 20 μm.

In Figure3 we show images from samples of mouse lung and brain, and an Arabidopsis root tip, taken with SBF-SEM. A single 2D image from a series shows the presence of immune cells in the alveoli of the mouse lung (Fig.3A). A reconstruction of 102 consecutive images adds context and geometric information (Fig.3B). The 3D presentation of a series of images shows how the immune cells are adhering to the alveoli while patrolling the airways. The extent of contact between the cell and the alveolar wall was not clear in the 2D sections and this example clearly demonstrates the value of 3D volume information. The strength of SBF-SEM is its ability to provide high-resolution images in *X*, *Y* and also image very large volumes of samples. The imaging of large fields of view at high *X*, *Y* resolution allows for zooming in on areas of interest in the large datasets. In Figure3C, a field of view of 25.4 × 25.4 μm^2^ of mouse brain tissue was imaged at 4000 × 4000 pixels, yielding 6.3-nm pixels, and block slicing was done at 40 nm. The overview image shows a neuronal cell body and the surrounding neuropil, including the Golgi apparatus in the cell body and synapses with clearly visible synaptic vesicles in the neurites. These high-resolution images allow for reconstructions of synaptic vesicles that have a size of 40–50 nm (Fig.3D). In Figure3E we show an Arabidopsis root tip sectioned with transverse slices of 75 nm, starting at the tip and continuing for 2000 images (150 μm). The image shows an overview of the whole diameter of the root (70 μm) from the tip comprising the columella, lateral root cap, quiescent centre and part of the meristem. Although the tip was sectioned transversally digital reslicing of the 3D image allows for examining any orientation that is desired. We used a similar dataset to zoom in and crop out a small area of 10.35 × 7.23 × 3.9 μm^3^. The dataset consisted of 6 × 6 × 25 nm^3^ voxels and in the images plasmodesmata, cytoplasmic channels that cross the cell wall, were visible as electron dense objects (Fig.3F). Using 3D volume representation and intensity threshold filtering, we can display the presence of plasmodesmata between cells, their distribution and relative positions. The complexity and number of plasmodesmal connections was not apparent in 2D representations (Zhu & Rost, 2000). The advantage of SBF-SEM is that it collects data that result in both overview and detail information.

**Figure 4:**
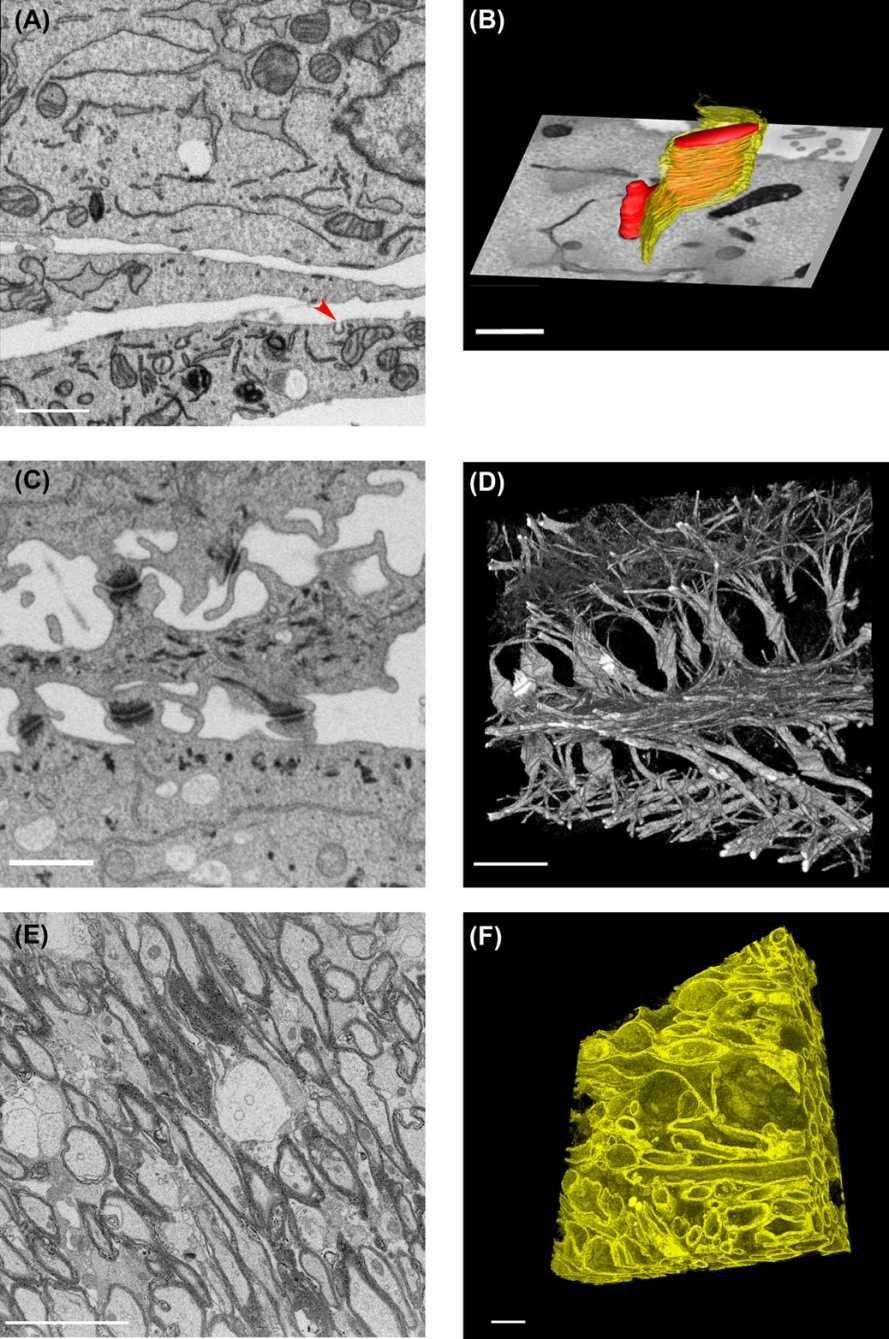
Examples of 3D-EM using FIB-SEM. (A) Mouse embryonic fibroblasts. A single image showing numerous subcellular organelles. The red arrowhead indicates a clathrin coated pit. Scale bar: 1 μm. (B) Manual segmentation of a dataset using IMOD consisting of 7,55 × 7,55 × 10 nm voxels. Reconstruction used to show the detailed geometry of the ER (yellow) and mitochondria (red). Scale bar: 1 μm. (C) Cultured lung epithelial cells from a dataset consisting of 6,2 × 6,2 × 15 nm voxels. A single image showing staining of intermediate filaments and desmosomes. Scale bar: 1 μm. (D) 3D volume rendering was done in Fiji showing the organization of the intermediate filaments and connections to the desmosomes. Scale bar: 1 μm. (E) Mouse brain tissue (corpus callosum) imaged with isotropic voxels (25 × 25 × 25 nm) from a dataset of 25 × 19 × 19 μm. Scale bar: 5 μm. (F) Volume reconstruction of a subset of the data (17 × 17 × 6,9 μm) was done in Fiji showing myelin sheaths running through the tissue. Scale bar: 5 μm.

FIB-SEM permits thinner *Z* slicing, because the technique allows for very precise sectioning in the nanometre range. Because of that reason it is very well suited for obtaining 3D volume images with small isotropic voxels (See Fig.1D). Making use of a Zeiss Auriga FIB-SEM we imaged mouse embryonic fibroblast cells, at a magnification and resolution that allows clear visualization of the endoplasmic reticulum (ER), mitochondria, plasma membrane, Golgi stacks, microtubules, endosomes and nuclear pores (Fig.4A). To visualize the 3D structure of the ER and mitochondria, segmentation was performed manually on the collected 2D images, which were then used for 3D rendering of the segmented objects. Figure4B shows part of a mitochondrion in red and the ER in yellow, in respect to an orthogonal *X*, *Y* view. This kind of visualization allows the precise geometry of complex structures like the ER to be visualized. To avoid time-consuming manual segmentation, acceptable segmentation can often be achieved by applying simple intensity threshold filters for structures that are more electron dense than their surroundings. In FIB-SEM images of cultured lung epithelial cells (Fig.4C) the desmosomes and intermediate filaments are more contrasted, compared to the other objects in the image. Visualizing only voxels with specific grey-values clearly shows the organization of the cell adhesion structures and how they are connected to the cytoskeleton (Fig.4D). FIB-SEM is the method of choice when detailed reconstructions are essential. In addition to cellular monolayers, we have also imaged tissues, more specifically mouse brain corpus callosum, containing mainly myelinated axons (Fig.4E). Here again structures with more contrast, in this case the myelin sheath surrounding the axons, can easily be segmented by using intensity threshold filtering, resulting in a 3D representation of the myelin ensheathments (Fig.4F).

### Optimizing staining and sample preparation

**Figure 5:**
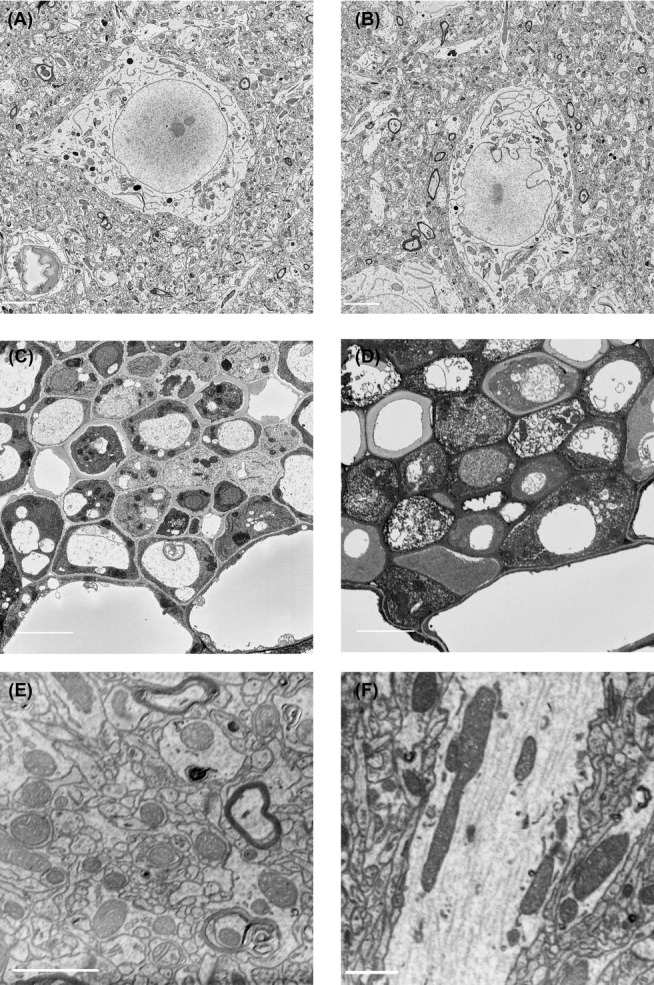
Developing new protocols for SBF-SEM. (A) Mouse brain tissue stained with an Osmium Thiocarbohydrazide Osmium protocol using Uranyl Acetate *en bloc* staining (Deerinck *et al*., 2010). Scale bar: 10 μm. (B) Mouse brain tissue stained as panel A, replacing uranyl acetate with lanthanide salts. Scale bar: 10 μm. (C) *Arabidopsis thaliana* root tip that was *en bloc* stained with uranyl acetate. Scale bar: 5 μm. (D) *Arabidopsis* root tip done under same conditions as panel C, except replacing uranyl acetate with lanthanide salts. Scale bar : 5 μm. (E) Mouse brain tissue in which contrast staining and embedding was performed using a microwave oven. imaged in a Zeiss Merlin with a Gatan Digiscan II detector. Scale bar: 1 μm. (F) A similar sample to panel E was imaged in a Zeiss Auriga using a BSE detector. Scale bar: 1 μm.

One significant difference between preparing samples for TEM and block-face imaging SEM is the need for more intense staining both to produce sufficient image contrast and also to make the sample more conductive. Several protocols have been published but individual optimizations for diverse samples such as yeast, viruses, plants, bacteria and even different mammalian tissues can yield improved results (Leser *et al*., 2009; Knott *et al*., 2011). Since the contrast in SBF-SEM is not dissimilar to TEMs used in the 1950’s and 1960s the older EM literature can be an extremely valuable source for testing and reintroducing steps to improve specimen fixation and contrast. For plant material we tested the use of ruthenium red that allowed us to better visualize plasmodesmata in the cell wall (Figs 3E and F). Replacing uranyl acetate by lanthanide salts (LS) yielded more cytoplasmic detail and contrast in several samples (Figs.5A and B) and also appeared beneficial in plant roots to produce darker staining of the cell wall (Figs.5C and D) which can be very useful in intensity threshold filtering based image segmentation although with some loss in cytoplasmic detail. Other older stains and fixatives are being investigated such as tannic acid, malachite green, and cuprolinic blue, which may come back into fashion as 3D SEM further develops.

A significant challenge when using the complicated protocols required for 3D block face imaging studies is the time required to process samples and embed them in plastic. In addition, different cells and tissue types can require additional steps to produce optimum results. Adding multiple staining steps and dealing with tissues that are difficult to infiltrate (such as plant and skin) can make the staining, dehydration and embedment protocols require 5–7 days. Thus, trying to develop optimal protocols can be time consuming. However, we have preliminary data that indicate that the use of a specially designed and regulated microwave oven (BioWave) for 3D SEM sample preparation may shorten the time for testing different staining and embedding conditions from multiple days to a few hours. In Figures5E and F we show two samples of brain prepared using microwave processing which took less than 6 h from fixation to polymerized plastic resin. The staining of the tissues and the infiltration and polymerization of the plastic appear identical to those samples processed conventionally. This method can potentially make the optimization of staining protocols for new tissue types much more efficient. It was equally successful for samples of mouse skin and *Arabidopsis* leaf (data not shown).

### Using both 3D SEM techniques on the same sample

To increase both the field of view and the resolution in three dimensions, a combination of SBF-SEM (largest field of view) and FIB-SEM (finest axial resolution) in the same sample is possible by transferring samples between SBF-SEM and FIB-SEM systems. Both technologies require samples that are *en bloc* contrast stained and plastic embedded, but there can be a difference in contrast in the images due to differences in sensitivity between detectors. The 3View detector allows image acquisition of a large field of view at high pixel resolution and is generally used at lower magnification. In FIB-SEM, smaller areas are imaged and the SEM is used at higher magnification allowing the backscatter detector to collect more electrons resulting in a better signal-to-noise ratio.

**Figure 6:**
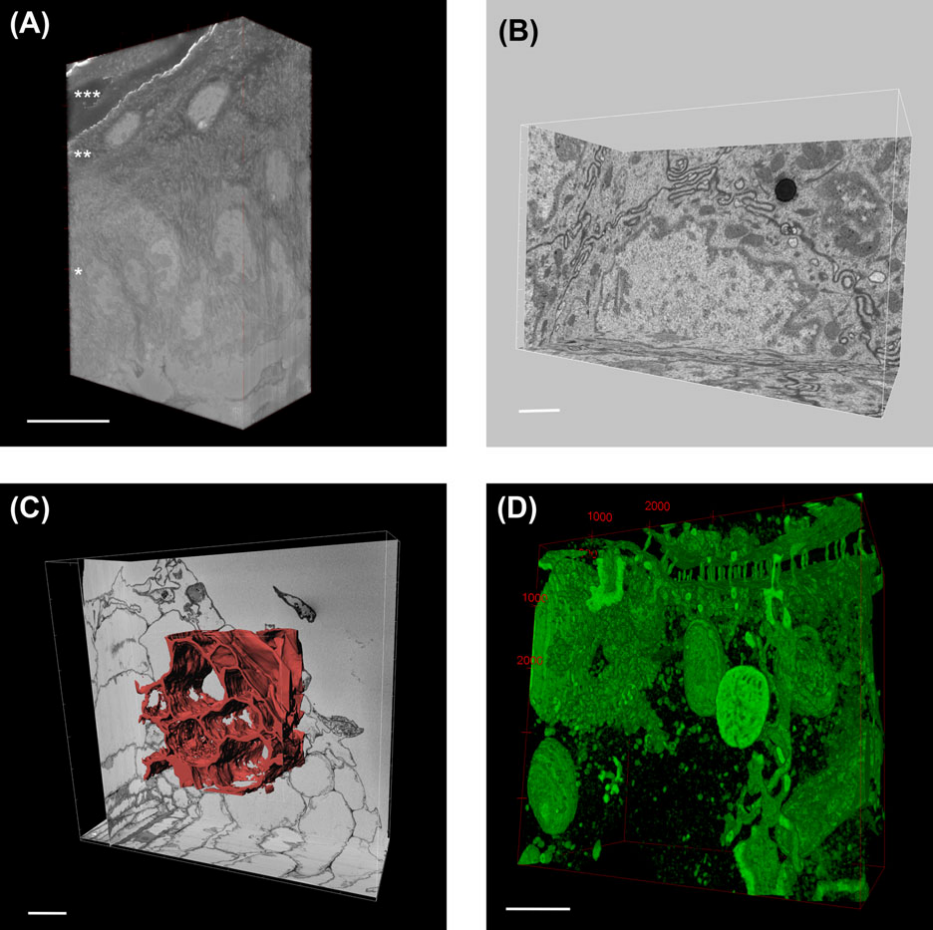
Consecutive SBF-SEM and FIB-SEM imaging. (A) A volume of 26,75 × 38,20 × 12,83 μm of normal mouse skin reconsted from SBF-SEM imaging. Undifferentiated basal layer (*) and differentiated layers (**granular layer and *** cornified layer) can be distinguished. Scale Bar: 10 μm. (B) A similar sample was used for imaging in a FIB-SEM. A basal layer keratinocyte is shown where subcellular organelles and connections between cells, such as desmosomes, are visible. Scale Bar: 1 μm. (C) Medicago truncatula root tips imaged by SBF-SEM. Orthogonal views of a volume of 177 × 166 × 60 μm consisting of 600, 100 nm slices is shown. Segmentation in red shows the cell walls and gives an overview on the general organization of the cells within the root tip. Scale Bar: 20 μm. (D) The same sample was transferred to a Zeiss Auriga FIB-SEM at 5 nm isotropic voxels. A selected region of 8 × 8 × 3 μm is shown in a 3D representation showing mitochondria, the Golgi apparatus, and plasmodesmata connecting to the ER. Scale Bar: 1 μm.

We have compared several samples in a Merlin SEM with 3View and Auriga FIB-SEM and noticed that in general sample preparation for FIB-SEM requires less metal staining to generate optimal contrast than is needed for SBF-SEM; however, samples can be successfully imaged even using the same preparation method. By using SBF-SEM on mouse skin we could image the different epidermal layers and dermis, so that it is possible to visualize the architecture of the tissue (Fig.6A). For a detailed view of the 3D cellular ultrastructure we transferred the samples to the FIB-SEM allowing clear visualization of desmosomes and cellular cytoskeleton (Fig.6B). In a similar way we imaged the root tips of *Medicago truncatula*, first by SBF-SEM at a Z section thickness of 100 nm, which allowed us to look at the general morphology of the cells (Fig.6C). The cells mainly consist of a vacuole, whereas the cytoplasm, containing all the organelles occupies a small volume near the cell wall. The same sample was transferred to the FIB-SEM and using the last image of a stack that was collected by SBF-SEM for orientation we imaged an area containing cytoplasm bordering the junction of 2 cells. The reconstruction of the area that was imaged shows plasmodesmata, ER, mitochondria and Golgi apparatus, obtained by intensity threshold filtering of a 3D dataset of 5-nm isotropic voxels (see Fig.6D).

### Towards a workflow for 3D–3D correlative microscopy

There are many roads to CLEM and our goal is to be able to image the exact same region with both imaging techniques, so to find back the same ROI, and to do this with a minimum of manual, labour intensive, steps. Our aim is to establish an efficient workflow for CLEM from 3D LM to 3D EM. A confocal image can be acquired from photons that are localized several micrometres below the sample surface and whose resolution will be no greater than 50 nm. In 3D SEM, the image will come from the sample surface and the maximum resolution will be about 5 nm. This presents several challenges, the greatest of which is matching the origin of the photon to its corresponding EM location. Ultimately this process has to deal with the resolution difference as well as the changes that a particular sample undergoes when being fixed, stained, dehydrated and embedded for EM, (cryo techniques used in several laboratories may be helpful in this regard as well as preserving a more natural structure). Until we can develop a cellular positioning system (CPS), which will allow us to precisely overlay the LM and EM datasets, we are restricted to cruder yet still effective means.

**Figure 7:**
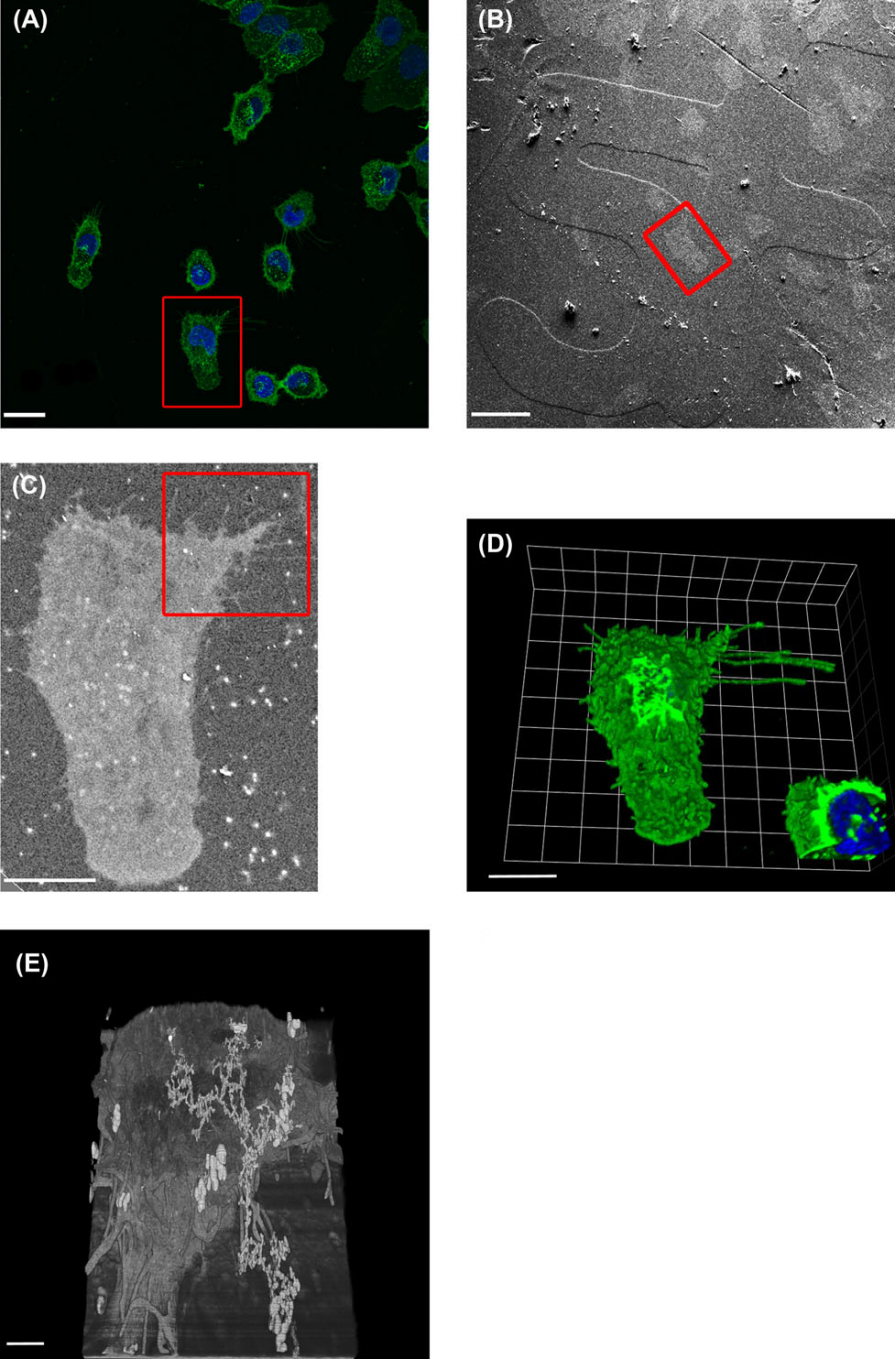
CLEM. (A) Human lung epithelial A549 cells were grown on gridded coverslips and imaged by confocal microscopy, with wheat germ agglutinin in green and nuclear dye in blue the cells of interest are highlighted in the red box. Scale bar: 24 μm. (B) An SEM image, using SE2 detector shows the lines of the coverslip grids. Scale bar: 50 μm. (C) An image of the block-face was taken at 15 kV, using a BSE detector confirming the identification of the cell of interest. The red box indicates the area that will be imaged with FIB-SEM. (D) 3D reconstruction of a confocal z-stack, showing the cell of interest. Scale bar: 10 μm. (E) 3D reconstruction of the FIB-SEM image of the area indicated with a red box in panel C. The imaged volume dimensions are 16,92 × 11,47 × 9,58 μm. Fiji was used for image processing. Scale bar: 20 μm.

For 3D CLEM it is crucial to develop a strategy for identifying the ROI imaged in the LM so that we can position the sample appropriately for 3D-SEM imaging. For cultured cells growing in monolayers, a method has been used for some time in which gridded cover slips can be used for LM making the cells of interest easily reacquired in the SEM (Polishchuk *et al*., 2000; Brown *et al*., 2009; Kobayashi *et al*., 2012). An example of this method generated in our laboratory can be seen in Figure7. After acquiring a confocal image and recording in which exact grid the cell of interest was located (Fig.7A), we processed the sample for FIB-SEM. We could retrace the right position, because the grid was printed into the resin and visible using a secondary electron detector (see Fig.7B). A high voltage back-scattered electron image of the block-face shows that the morphological features of the cell were identical to the features that were visualized by confocal microscopy (Figs.7A and C). In Figures7D and E we show a confocal image and 3D-SEM image of the same cell.

**Figure 8:**
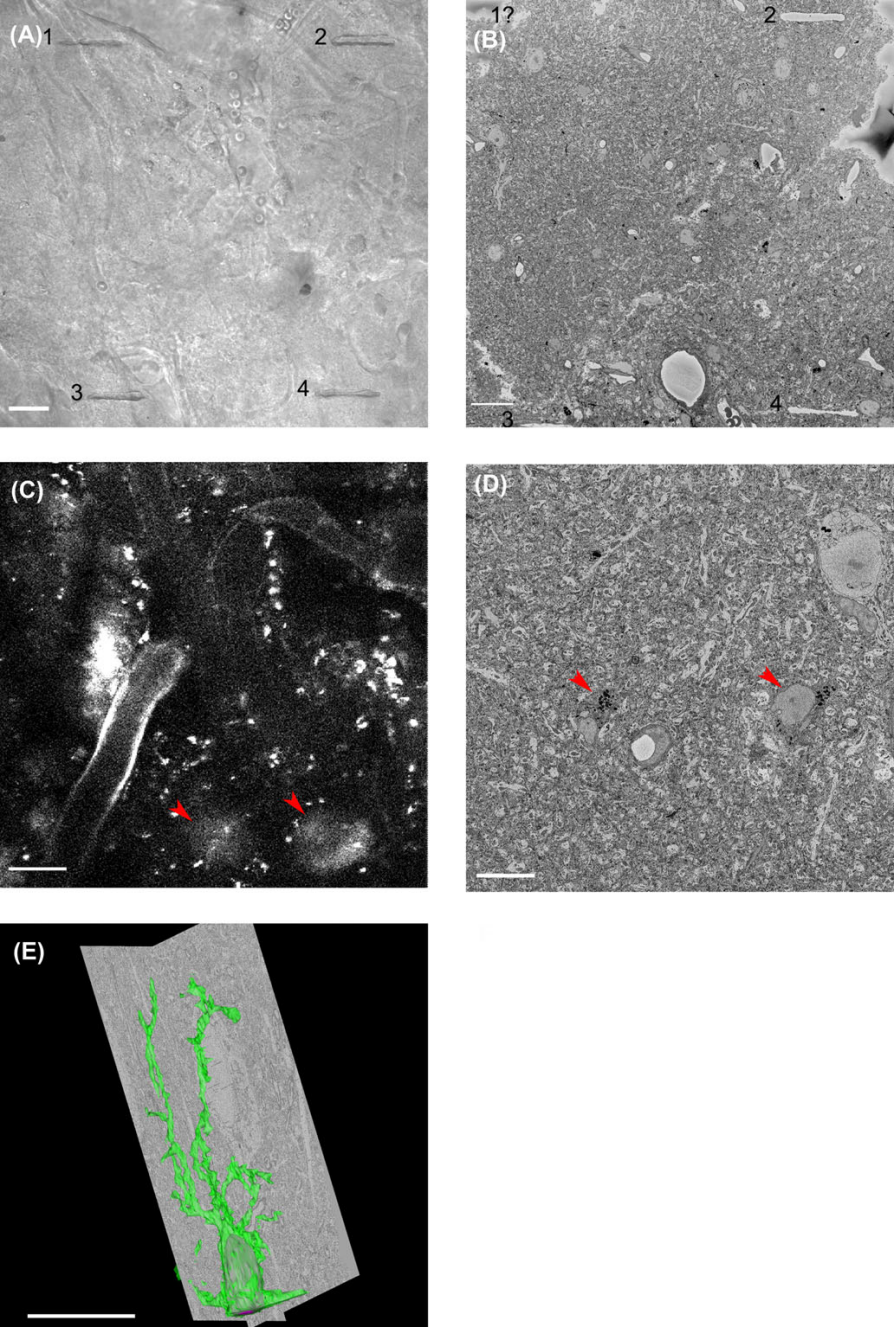
CLEM. (A) NIRB was used to introduce scars in brain tissue, after live-cell imaging, in order to create reference points that allow correlation of the LM images to SBF-SEM data. A bright-field image shows the presence of four laser-brands (1–4). Scale bar: 20 μm. (B) The first image of a stack, generated by SBF-SEM, shows laser-brands 2 and 4 in the corners. Importantly, no apparent damage to the ultra-structure surrounding the brands is visible. Scale bar: 10 μm. (C) A confocal image showing *in vivo* imaging of astrocyte Ca^2+^ activity. Red arrows indicate two areas of astrocyte activity a few microns below the laser-brands. Scale bar: 30 μm. (D) Based on the laser-brands, the two astrocytes, indicated in panel C, could be retraced in the SBF-SEM data-set. Arrowheads indicate the astrocytes in a single SEM image. Scale bar: 10 μm. (E) Manual segmentation of the astrocytes was done using IMOD. 2 orthogonal views and the segmented astrocyte in green are shown in the volume. Scale bar: 10 μm.

Another method to identify a ROI between LM and EM data sets was developed by Bishop *et al*. (2011). Near-infrared laser branding (NIRB) is a method that allowed introduction of markers around a ROI with the use of an infrared laser that brands scars into tissue. The technique was used by Maco *et al*., to perform 3D CLEM, combining live imaging of axons and dendrites in mouse brain with FIB-SEM (Maco *et al*., 2013). We made use of NIRB in combination with morphological features for CLEM on mouse brain. First Ca^2+^ activity in specific astrocytes was imaged *in vivo* several hundreds of microns below the surface of the brain. After fixation and sectioning we were able to relocate the area of interest, using the pattern of blood vessels as reference points (Knott *et al*., 2009; Maco *et al*., 2013). Four small marks were branded surrounding this area (Fig.8A). The section was then stained and embedded for SBF-SEM. Using the SBF-SEM, we approached 1 μm at a time (in 100 nm steps) until the marks were seen (Fig.8B). With the marks delineating the edge of the field of view we reacquired the astrocytes of interest just below the marks (Figs.8C and D). Part of an astrocyte was reconstructed using IMOD (Fig.8E).

## Discussion and future prospects

The use of volume SEM in Life Sciences is dramatically increasing, for both SBF-SEM and FIB-SEM, and these techniques are being applied to many diverse samples. Both SBF-SEM and FIB-SEM have distinct advantages and share some commonalities in aspects of sample preparation, imaging parameters and data analysis. The major difference is in the *Z* resolution that the systems can provide. With FIB-SEM small isotropic voxels are achievable but only in relatively small volumes. SBF-SEM can handle much larger volumes but is limited in *Z* resolution. Ideally, a laboratory or core facility performing volume SEM would benefit from having access to both types of devices even though the cost and complexity of using both is significant.

Although many of the original biological applications for SBF-SEM are in the field of neuroscience and protocols were optimized for brain samples (Young *et al*., 1993; Ballerini *et al*., 2001; Denk & Horstmann, 2004; Drobne *et al*., 2004; Drobne *et al*., 2005; Heymann *et al*., 2006, Knott *et al*., 2011). To some extent, FIB-SEM is more forgiving than SBF-SEM and in some cases samples prepared for TEM will give acceptable (yet less than optimal) results. As 3D SEM techniques become more available examples are appearing of the use of both techniques in other cells and tissues (Pollier *et al*., 2013; Fendrych *et al*., 2014; Peddie & Collinson, 2014; Rybak *et al*., 2014). However, in many laboratories, sample preparation for brain tissue has been used as a basis for preparing other tissue types for 3D SEM. This is not optimal, especially in advanced imaging cores that are confronted with requests to image a wide range of different tissues or species. Because sample preparation and sample handling is very time-consuming, and in view of the fact that the 3D EM field is developing rapidly, staining and embedding protocols are often copied without thorough optimization for the particular tissue. Ideally, for each species/tissue type a unique sample preparation protocol should be established. In Table1 we provide an overview of different tissues that we have imaged so far by SBF-SEM or FIB-SEM. For all these a separate staining and embedding protocol was developed to optimize the subsequent imaging. Because the SEM image is generated by a backscattered electron detector, it is crucial that samples are stained with several heavy metals that provide both contrast in the image and conductivity of the sample, to help prevent charging. Generally, *en bloc* staining will consist of (multiple) osmium, uranyl acetate and sometimes Pb infiltration steps (Deerinck *et al*., 2010; Joensuu *et al*., 2014). Besides changing incubation times with osmium, Pb and uranyl acetate or Lanthanide salts, it could be important to reintroduce other and older EM stains. Other helpful procedures such as protecting fragile samples from mechanical damage during the extensive processing required are also important. We adapted our protocol, based on a publication to prepare root tips for TEM (Wu *et al*., 2012). Before the first osmium step, we surround these samples in agarose to protect the sample from collapsing or mechanical stress and this will also improve conductivity because osmium binds the agarose. In future it would be helpful to create a publicly accessible Web resource to collect and make available protocols used for different sample types. Volume SEM is still a developing field and ensuring that protocols are optimized for each sample will help demonstrate its potential to produce high quality 3D data sets.

Our results from many diverse samples have shown the degree of detail that can be achieved in volume SEM. Features such as *Arabidopsis* plasmodesmata which appeared as sparse structures in 2D TEM were shown to be extensive and complex. Reconstructions of entire root cap cells in this same species would have been impossible in single TEM sections because of their morphology which wraps them in a spiral pattern around the root tip allowing only a small subportion of the cells to be seen in single sections. Our SBF-SEM results allowed the entire rank of cells to be visualized greatly enhancing our understanding of their true morphology (Fendrych *et al*., 2014). Even structures which appeared relatively simple in 2D EM, such as mitochondria and ER, look much more complex when seen in their full 3D geometry. Just as the full extent of the branches of a tree cannot be appreciated from a single photograph, the real complexity becomes apparent when you can walk around it and see its full 3D shape. Similarly, the complexities and interrelationships of the ultrastructure in many cells and tissues will appear much more clearly when volume EM reconstructions are made.

The full potential that volume EM studies can bring to biological imaging cannot be overstated. Ultimately it is desirable to have the freedom to transverse the resolution scale from mm to nm to allow for a “Google Earth” view of cells and tissues. This not only opens up the possibility of identifying rare events which can be more easily located in a large overview image, but also the opportunity to zoom in on those events and see them at the scale of nanostructure. Volume EM data also opens up the possibility of quantitative EM studies without the restrictions of small sample size which 2D EM has mostly had to live with. Linking the functional information that can be obtained with live cell and tissue imaging and relating it to the underlying fine structure of the cell opens up powerful possibilities. The prospect of creating 3D nanostructural atlases of whole tissues or organisms, which has already been extensively discussed in the literature and is in process for such tissues as brain and *C. elegans* (Hall & Altun, 2008; Ellisman *et al*., 2011), presents another new opportunity to understand life at levels heretofore difficult or impossible to achieve.

Volume EM data sets are extremely large – even with small samples – and the computing infrastructure necessary for simple visualizations and reconstructions is not yet efficient nor well integrated. Manual reconstructions of even small numbers of organelles (such as seen in Fig.4B) take many hours. In addition, the more difficult intensely laborious steps involved in accurately quantifying 3D EM data need to be improved. Because EM reveals the full ultrastructural details of a cell, data that has been used to answer one specific question can be reassessed. It is possible to conceive of data from a single cell or tissue type being reused again and again to answer different questions. A repository of 3D EM data sets could be an invaluable resource to scientists from many areas of interest. Thus, volume EM has the potential not just to answer the questions we have today, but also to be invaluable in answering questions we have not yet even come up with. However, to take advantage of these data, computing needs to catch up with imaging as our ability to collect data far exceeds our ability to analyse it.

Improvements in the entire workflow of 3D EM are necessary if the technique is to become fully integrated in life science research. 3D SEM is not a trivial technique technically, nor is it inexpensive. Therefore, it is essential that the entire process be made as efficient as possible. Optimal sample preparation procedures for different sample types, optimal resin formulations that work for both SBF- and FIB-SEM, and the optimal hardware parameters for imaging are still being developed. On the IT side, reconstructing high-quality images, automated segmentation of specific substructures from the rich data sets produced, easily and accurately correlating the 3D EM data with 3D LM data and archiving and data mining of existing data sets is even more in need of improvement. Nevertheless, this method opens up so many possibilities for seeing cellular ultrastructure in new ways it will certainly redefine the way we see the cellular nanoworld.
